# Does a "Level I Evidence" rating imply high quality of reporting in orthopaedic randomised controlled trials?

**DOI:** 10.1186/1471-2288-6-44

**Published:** 2006-09-11

**Authors:** Rudolf W Poolman, Peter AA Struijs, Rover Krips, Inger N Sierevelt, Kristina H Lutz, Mohit Bhandari

**Affiliations:** 1 Department Surgery, Division of Orthopaedic surgery, McMaster University, Hamilton General Hospital, 7 North, Room 727, 237 Barton Street East, Hamilton, Ontario L8L 2X2, Canada; 2 OrthoTrauma Research Centre Amsterdam, Department of Orthopedic Surgery, Academic Medical Centre, University of Amsterdam, G4 Noord, PO Box: 22660, 1100 DD, Amsterdam, The Netherlands; 3 Department of Orthopaedic Surgery and Traumatology Ziekenhuis Hilversum, Postbus 10016, 1201 DA, Hilversum, The Netherlands; 4 Department of Clinical Epidemiology and Biostatistics, McMaster University, Hamilton General Hospital, 7 North, Room 727, 237 Barton Street East Hamilton, Ontario L8L 2X2, Canada

## Abstract

**Background:**

The Levels of Evidence Rating System is widely believed to categorize studies by quality, with Level I studies representing the highest quality evidence. We aimed to determine the reporting quality of Randomised Controlled Trials (RCTs) published in the most frequently cited general orthopaedic journals.

**Methods:**

Two assessors identified orthopaedic journals that reported a level of evidence rating in their abstracts from January 2003 to December 2004 by searching the instructions for authors of the highest impact general orthopaedic journals. Based upon a priori eligibility criteria, two assessors hand searched all issues of the eligible journal from 2003–2004 for RCTs. The assessors extracted the demographic information and the evidence rating from each included RCT and scored the quality of reporting using the reporting quality assessment tool, which was developed by the Cochrane Bone, Joint and Muscle Trauma Group. Scores were conducted in duplicate, and we reached a consensus for any disagreements. We examined the correlation between the level of evidence rating and the Cochrane reporting quality score.

**Results:**

We found that only the Journal of Bone and Joint Surgery – American Volume (JBJS-A) used a level of evidence rating from 2003 to 2004. We identified 938 publications in the JBJS-A from January 2003 to December 2004. Of these publications, 32 (3.4%) were RCTs that fit the inclusion criteria. The 32 RCTs included a total of 3543 patients, with sample sizes ranging from 17 to 514 patients. Despite being labelled as the highest level of evidence (Level 1 and Level II evidence), these studies had low Cochrane reporting quality scores among individual methodological safeguards. The Cochrane reporting quality scores did not differ significantly between Level I and Level II studies. Correlations varied from 0.0 to 0.2 across the 12 items of the Cochrane reporting quality assessment tool (p > 0.05). Among items closely corresponding to the Levels of Evidence Rating System criteria assessors achieved substantial agreement (ICC = 0.80, 95%CI:0.60 to 0.90).

**Conclusion:**

Our findings suggest that readers should not assume that 1) studies labelled as Level I have high reporting quality and 2) Level I studies have better reporting quality than Level II studies. One should address methodological safeguards individually.

## Background

The International Society of Medical Editors emphasises the importance of effective reporting in medical literature [1,2]. However, previous studies have identified poor quality of reporting of study methodology in the orthopaedic literature [3,4].

Since January 2003, all clinical scientific articles published in the American Volume of The Journal of Bone and Joint Surgery (JBJS-A) have included a level of evidence rating [5,6]. The Levels of Evidence Rating System is a tool that classifies the quality and design of a study. Based on a review of several existing evidence rating systems [5,6], JBJS-A has designed a scheme that uses five hierarchical levels for each of the four different study reporting types (therapeutic studies, prognostic studies, diagnostic studies, and economic and decision analyses). According to the Levels of Evidence Rating System hierarchy, randomised controlled trials (RCTs) occupy the top positions (Level I & Level II evidence) and expert opinion lies at the bottom (Level V evidence). Previous research has suggested that investigators with training in epidemiology can achieve nearly perfect agreement when applying the Levels of Evidence Rating System to a study [7]. This research suggests reliability; however, the system's validity remains debatable [7].

The Levels of Evidence Rating System causes readers to infer that Level I evidence RCTs are of better methodological quality than Level II evidence RCTs [8]. The Editorial Board Members of the JBJS-A reported that the Levels of Evidence Rating System would have important advantages such as enabling the journal "to monitor and to periodically report trends in the quality of orthopaedic clinical research" [5]. Furthermore, the editors wrote that "higher levels of evidence should be more convincing to surgeons attempting to resolve clinical dilemmas" [5].

The assessment of the true quality of published studies remains challenging [9-11]. One can judge the true study quality only if the reporting of the trial is done in a clear and comprehensive manner. For example, in some published articles within Internal Medicine literature, the authors failed to report important methodological safeguards that were in fact used during the conduct of the trial [12]. Therefore, high quality depends not only on the nature of the work, but also on the completeness of the reporting [2]. Most readers of medical literature will base their assessment of study quality solely on the information contained in the report of a trial, as they will not be bothered to contact the author for additional information [12].

The most developed criteria for guiding clinicians in their assessment of study reporting quality have been proposed for RCTs, since RCTs are a study design that yield the lowest chance of bias [11,13]. The Consolidated Standards for Reporting of Trials (CONSORT) statement was developed to help authors present their trial in a structured and complete manner. Assessors, on the other hand, use different tools to assess the quality of a trial. The Cochrane Collaboration, which is the largest database of systematic reviews (N = 4041, October 2005) and clinical trials (N = 454449, October 2005) in existence, has adopted one commonly utilized rating system to guide assessors in their assessment of study quality, as evaluated through the information contained in the report [9,14].

Given the upcoming use of the Levels of Evidence Rating System in orthopaedic literature, we aimed to evaluate the reporting quality of RCTs published in the JBJS-A from 2003 to 2004 (Level I and Level II evidence ratings). We, therefore, extracted the level of evidence rating as published in each RCT and compared this rating with the well-established Cochrane Bone, Joint and Muscle Trauma Group's reporting quality assessment tool. We chose the JBJS-A because it was the most frequently sited general orthopaedic journal (ISI web of science), and the only journal that used this Levels of Evidence Rating System in the eligible time period.

Our hypotheses were twofold: 1) Level I evidence studies in a high impact general orthopaedic journal would not necessarily have high quality reporting and 2) the reporting quality of RCTs would not differ among trials labelled as Level I or Level II evidence.

## Methods

### Study design

We conducted a methodological study. We assessed the level of evidence rating assigned to a series of RCTs with the Cochrane reporting quality score.

### Eligibility criteria

Two assessors (RWP, MB) identified orthopaedic journals that reported a level of evidence rating in their abstracts from January 2003 to December 2004 by searching the instructions for authors of the highest impact general orthopaedic journals (JBJS-A, JBJS-British Volume, Clinical Orthopaedics and Related Research, and Acta Orthopaedica). Within the eligible journal, two assessors (RWP, RK) hand searched all issues from 2003–2004. The eligibility criteria were determined and set a priori. Eligible studies included those reported as RCTs involving a therapeutic intervention and using human subjects. We conducted searches in duplicate, and the consensus of three authors (RWP, RK, MB) resolved any disagreements.

### Study demographic information

The relevant demographic information was extracted from each eligible study by one investigator (RWP) and rechecked for accuracy by a second investigator (PAAS). The extracted data included (1) first author (surgeon, non-surgeon, or epidemiologist), (2) cited statistical support or methodological support by a department of clinical epidemiology or public health, (3) year of publication, (4) total sample size, (5) number of centres, (6) name of intervention, (7)category of intervention (fracture treatment, treatment of degenerative disease of the spine and joints, drug trial, pain management, or other), (8) body region (upper extremity, long bones of lower extremity, spine, hip and knee, or foot and ankle, DVT, or other),(9) financial support (yes or no), (10) direction of results(positive [if the findings of the randomised trial were significant]or negative [if they were not significant]), and (11) trial reported according to the CONSORT statement (yes or no).

### Levels of evidence

One of the authors (RWP) extracted the level of evidence from each abstract of the included RCTs. A second author (INS) double-checked the evidence rating to ensure that it was correctly extracted from the paper.

### Quality of reporting assessment

Two authors (RWP, PAAS), blinded to study author and institution, graded the reporting quality of the included RCTs using the Cochrane reporting quality assessment tool, which was devised by the Cochrane Bone, Joint and Muscle Trauma Group, formally known as the Musculoskeletal Injuries Group. This scoring scheme covers aspects of internal and external validity for the assessment of methodological quality [15]. We used this reporting quality assessment tool as our reference standard due to its widespread use [15] and association with the methodologically rigorous Cochrane reviews of RCTs [9,16,17]. The tool consists of twelve items important for the critical appraisal of a RCT report. A coding manual was available from the group's website [15]. The highest possible score for each item was 2 and the lowest was 0. Additional file 1 contains the scoring system that we used to identify the important aspects of reporting methodological quality [see Additional file 1]. We followed therecommendation found in the Cochrane Handbook which stated that at least two authors assess information that involves subjective interpretation and information that is critical to the interpretation of results (e.g., outcome data) [18].

Studies that randomly allocated patients (Item D), concealed randomisation (Item A), blinded participants (Items C, E, F) and documented study withdrawals (Item B) were reported to reflect higher quality [19,20]. We scored all reported methodological safeguards separately for all identified RCTs. Different quality aspects can be weighted differently and thresholds are arbitrary [10]; therefore, we did not summarize the scores in totals, but reported the raw data.

### Ensuring the accuracy of the quality rating

We used Intraclass Correlation Coefficients (ICC) to measure the agreement between the assessors' assessment of study reporting quality. We used Landis and Koch's suggested criteria for the interpretation of the agreement: 0 to 0.2 represented slight agreement, 0.21 to 0.40 fair agreement, 0.41 to 0.60 moderate agreement, and 0.61 to 0.80 substantial agreement. A value above 0.80 was considered almost perfect agreement [21]. Regardless, if two assessors disagreed even slightly, consensus was attempted after carefully reading the article a second time in a consensus meeting. In situations where discrepancies persisted despite a consensus meeting, a third assessor was asked for an opinion on the specific item to reach final consensus. This method of quality assessment a final consensus meeting has been commonly used in Cochrane reviews. All assessors (RWP, PAAS, MB, and RK) were well trained in quality assessments, were clinically active in orthopaedic surgery, had completed a Cochrane Review course, and had co-authored in Cochrane systematic reviews of RCTs.

### Statistical analysis

Data was analysed using the SPSS statistical software package (version 11.2; SPSS, Chicago, Illinois). We summarized all individual Cochrane reporting quality items with mean scores, which we then compared with student t-tests. We compared more than two means with single factor analysis of variance adjusted for post-hoc comparison testing. We then compared the total scores (0–2) for each item in the Cochrane reporting quality assessment tool with the level of evidence rating as published in JBJS-A. Prior to the analysis, we identified Cochrane Items A, C, E, F, and L to be most similar to the description of the levels of evidence. In a subgroup analysis, we compared the levels of evidence as described in the instructions for authors with the Cochrane reporting quality items that were deemed similar (Table 1). We used the Spearman's correlation (non-parametric test, non-normally distributed data) to calculate the correlation between the JBJS-A level of evidence rating and the total Cochrane reporting quality score, and the correlation between the JBJS-A level of evidence rating and Items A, C, E, F, and L of the Cochrane reporting quality score. For correlations, we categorized the levels of evidence from 1 to 4 (1 = level 1A, 2 = level 1B, 3 = level 2-1, 4 = level2-2) with 1 representing the highest level. We used p < 0.05 to represent statistical significance. All tests of significance were two-tailed.

**Table 1:** Cochrane Items Closely Related to the Levels of Evidence

Levels of Evidence JBJS in instruction for Authors		Cochrane Item											
Level	Description	A	B	C	D	E	F	G	H	I	J	K	L

I	High-quality randomised controlled trial with statistically significant difference or no statistically significant difference but narrow confidence intervals	2	X	2	x	2	2	x	x	x	x	x	2
II	Lesser-quality randomised controlled trial (e.g., <80% follow-up, no blinding, or improper randomisation)	0	X	0	x	0	0	x	x	x	x	x	0

Levels of Evidence compared with separate Cochrane items. 2 is maximal possible Cochrane score. 0 is minimal possible score. X is not described in instruction for authors.

### Sample size

Our study sample size included all RCTs published in the JBJS-A from January 2003 to December 2004. We required at least 30 eligible RCTs to provide sufficient correlation data on the level of evidence ratings and the Cochrane reporting quality scores (alpha = 0.05, Beta = 0.20, rho_null _= 0.2, rho = 0.7).

## Results

### Study demographic information

Of the four high impact orthopaedic journals, only JBJS-A used the level of evidence rating from 2003 to 2004. We identified 938 publications in the JBJS-A from January 2003 to December 2004. Of these publications, 32 (3.4%) were RCTs that fit the eligibility criteria. Thirty (94%) of the first authors were surgeons and 2 (6%) were non-surgeons. In 5 (16%) of the RCTs, at least one author had cited training in biostatistics (MSc or PhD) or was affiliated with a department of statistics, public health, or clinical epidemiology. The 32 RCTs included a total of 3543 patients, with sample sizes ranging from 17 to 514 patients. Six (19%) of the studies were performed in two or more centres, 11 (34%) focused on interventions related to the treatment of degenerative joint disease, 7 (22%) focused on fractures, and the remainder involved problems affecting the upper extremity [5 (16%)], the foot and ankle [6 (19%)], and the knee [9 (28%)]. Four (13%) RCTs were reported according to the CONSORT statement (Table 2). References to the included studies can be found in Additional file 2 [see Additional file 2].

**Table 2:** Characteristics of the Thirty-two Trials

**Characteristics**	**No. of Studies (%)**
*Total No. of RCTs*	32 (100%)
*First author*	
Surgeon	31 (97%)
Nonsurgeon	1 (3%)
*Epidemiology affiliation*	
Yes	3 (9%)
No	29 (91%)
*Category of trial*	
Fracture treatment	7 (22%)
Treatment of degenerative disease	11 (34%)
Drug trial	4 (13%)
Pain management	0 (0%)
Other	10 (31%)
*Region of body*	
Upper extremity	5 (16%)
Lower-extremity long bones	2 (6%)
Spine	2 (6%)
Hip	5 (16%)
Knee	9 (28%)
Foot and ankle	6 (19%)
Soft tissue	2 (6%)
DVT	1 (3%)
*Number of centres*	
Single	26 (81%)
Multi	6 (19%)
*Funding received*	
Yes	17 (53%)
No	15 (47%)
*Direction of results*	
Positive	24 (75%)
Negative	8 (25%)
*CONSORT*	
Yes	4 (13%)
No	28 (87%)

### Levels of evidence

Of the 32 included RCTs, 29 were reported as Level I studies and 3 were reported as Level II studies. Level I studies were further subgrouped into 22 Level-1A and 7 Level -1B (RCT-no significant difference, but narrow confidence intervals) studies. Level II studies were also subgrouped into 1 Level II-1 and 2 level II-2 studies as extracted from the included papers' abstract.

### Limitations in quality of reporting (Hypothesis 1)

Only 12 (38%) of the 32 included RCTs clearly described allocation concealment (Item A). Seven (22%) clearly described an intention to treat analysis (Item B). Thirteen (41%) clearly described the blinding of outcome assessors (Item C). Twenty-three (72%) clearly described the comparability of the treatment and control group at entry (Item D). Six (19%) of the 32 RCTs clearly described the blinding of participants (Item E). Only 2 (6%) of the studies clearly described the blinding of treatment providers (Item F). Seventeen (53%) clearly described identical care programmes other than the trial options (Item G). Of the 32 RCTs, 25 (78%) adequately described the inclusion and exclusion criteria (Item H). Of all the items, I and J were described best in all 32 RCTs: 31 (97%) clearly described the interventions and 31 (97%) clearly described the outcome measures used. Twenty-two (69%) clearly described a useful diagnostic test in the outcome assessment (Item K). Only 10 (31%) described an appropriate duration of follow-up (Item L).Table 3 shows all data for each RCT.

**Table 3:** Cochrane Bone, Joint and Muscle Injury Group scores for all 32 RCTs.

**Study**	**Level of Evidence**	**Cochrane Bone, Joint and Muscle Trauma Group reporting quality assessment score item (see below)**											
		**A**	**B**	**C**	**D**	**E**	**F**	**G**	**H**	**I**	**J**	**K**	**L**

1	II-2	0	0	0	0	0	0	2	2	2	1	2	1
2	I-1b	1	1	2	2	0	0	2	2	2	2	2	2
3	I-1a	1	0	2	2	2	0	1	2	2	2	1	1
4	I-1a	1	1	0	2	0	0	2	2	2	2	1	1
5	II-2	2	1	0	0	0	0	0	2	2	2	2	2
6	I-1b	2	0	2	2	0	0	0	2	2	2	2	1
7	I-1a	1	0	0	2	0	0	2	2	2	2	2	0
8	I-1a	1	2	0	2	0	0	1	2	2	2	2	2
9	I-1a	2	0	0	2	0	0	1	1	2	2	2	2
10	I-1a	1	0	0	2	0	0	2	1	2	2	1	1
11	I-1b	1	0	2	0	0	0	2	1	2	2	1	1
12	I-1a	1	2	0	1	0	0	1	1	1	2	2	2
13	I-1a	2	0	1	2	0	0	0	1	2	2	1	1
14	I-1a	0	1	0	2	0	0	1	2	2	2	2	1
15	I-1a	2	1	0	2	0	0	1	2	2	2	2	1
16	I-1a	1	1	0	1	0	0	0	1	2	2	2	1
17	I-1b	0	0	0	0	0	0	0	2	2	2	1	1
18	I-1a	0	1	2	0	0	0	2	2	2	2	2	0
19	I-1a	2	1	2	2	0	0	2	2	2	2	2	2
20	I-1a	1	2	0	2	2	0	2	2	2	2	2	1
21	I-1a	1	0	2	2	0	0	2	2	2	2	2	2
22	I-1a	1	1	0	0	0	0	2	2	2	2	2	2
23	I-1a	2	2	0	2	0	0	2	2	2	2	2	2
24	I-1a	2	1	2	1	2	2	2	2	2	2	2	1
25	I-1b	0	2	2	2	0	0	2	2	2	2	2	1
26	I-1a	2	0	2	2	2	0	0	2	2	2	2	2
27	I-1b	2	2	2	2	2	0	2	2	2	2	2	1
28	I-1a	2	1	2	2	0	0	0	2	2	2	1	1
29	I-1a	2	0	0	2	0	0	2	2	2	2	1	1
30	I-1a	1	1	0	2	0	0	0	1	2	2	1	1
31	I-1b	1	2	2	2	2	2	2	2	2	2	2	1
32	II-1	1	1	0	2	0	0	0	2	2	2	1	1
		**A**	**B**	**C**	**D**	**E**	**F**	**G**	**H**	**I**	**J**	**K**	**L**
**Number of studies with maximum score**		**12**	**7**	**13**	**23**	**6**	**2**	**17**	**25**	**31**	**31**	**22**	**10**
**Percentage of studies with maximum score (%)**		**38**	**22**	**41**	**72**	**19**	**6**	**53**	**78**	**97**	**97**	**69**	**31**

Cochrane Bone, Joint and Muscle Trauma Group reporting quality assessment items:

A. Was the assigned treatment adequately concealed prior to allocation?

B. Were the outcomes of participants who withdrew described and included in the analysis (intention to treat)?

C. Were the outcome assessors blinded to treatment status?

D. Were the treatment and control group comparable at entry?

E. Were the participants blind to assignment status after allocation?

F. Were the treatment providers blind to assignment status?

G. Were care programs, other than the trial options, identical?

H. Were the inclusion and exclusion criteria clearly defined?

I. Were the interventions clearly defined?

J. Were the outcome measures used clearly defined?

K. Were diagnostic tests used in outcome assessment clinically useful?

L. Was the surveillance active, and of clinically appropriate duration?

[See Additional file 1].

Among items closely corresponding to the Levels of Evidence Rating System criteria (Items A, C, E, F, and L), assessors achieved substantial agreement (ICC = 0.80, 95%CI:0.60 to 0.90). Across each of the 12 items, however, agreement varied (Range of ICC = 0 to 0.80). In all cases, assessors achieved consensus, either alone or with a third, intervening reviewer.

### Correlation between Cochrane reporting quality scores and reported levels of evidence (Hypothesis 2)

We compared the mean score in each item of the Cochrane reporting quality assessment tool separately (Items A through L) with each level of evidence (Table 4). Mean quality scores did not significantly differ across the 12 separate items of the Cochrane reporting quality assessment tool (Table 4). Correlations varied from 0.0 to 0.2 across the 12 items of the Cochrane reporting quality assessment tool (Table 4).

**Table 4:** Mean and median Cochrane score for all items compared with Levels of Evidence

Cochrane score (max = 2 points for each A-L)												
Level JBJS	A	B	C	D	E	F	G	H	I	J	K	L
% with score = 2	38%	22%	41%	72%	19%	6%	53%	78%	97%	97%	69%	31%
Mean Level I-1a	1.3	0.8	0.7	1.7	0.4	0.1	1.3	1.7	2.0	2.0	1.7	1.3
**Median Level I-1a**	**1.0**	**1.0**	**0.0**	**2.0**	**0.0**	**0.0**	**1.5**	**2.0**	**2.0**	**2.0**	**2.0**	**1.0**
Mean Level I-1b	1.0	1.0	1.7	1.4	0.6	0.3	1.4	1.9	2.0	2.0	1.7	1.1
**Median Level I-1b**	**1.0**	**1.0**	**2.0**	**2.0**	**0.0**	**0.0**	**2.0**	**2.0**	**2.0**	**2.0**	**2.0**	**1.0**
Mean Level II	1.0	0.7	0.0	0.7	0.0	0.0	0.7	2.0	2.0	1.7	1.7	1.3
**Median level II**	**1.0**	**1.0**	**0.0**	**0.0**	**0.0**	**0.0**	**0.0**	**2.0**	**2.0**	**2.0**	**2.0**	**1.0**
ANOVA (p value)*	NS	NS	NS	NS	NS	NS	NS	NS	NS	NS	NS	NS
Correlation (-)	0.04	0.2	0.1	0.2	0.1	0.2	0.0	0.3	0.1	0.2	0.03	0.1

p values corrected for post hoc comparisons (Bonferroni), NS = non-significant p value (P > 0.05)

## Discussion

### Summary of key study findings

The results of our methodological study demonstrated two key findings 1) Level I evidence studies revealed important limitations in their quality of reporting and 2) non- significant difference in the quality of reporting between studies labelled as Level I or Level II evidence.

### Strengths and weaknesses

Our study is strengthened by the use of a well-described and commonly used quality assessment tool from the Cochrane Collaboration that identifies the relevant methodological aspects of trials as reported and assesses these aspects individually. Furthermore, all assessors (RWP, PAAS, MB, RK) were well trained in quality assessments. Our decision to conduct assessments in duplicate (and triplicate when assessors disagreed) further strengthened the rigor of our assessments [18]. The paucity of Level II studies in our series limited inferences about the correlation data with level of evidence ratings. Our finding that the mean overall scores between Level I and Level II studies did not significantly differ was likely underpowered. The sample size calculation was difficult since clinicians have made arguments against calculating totals in quality scores (see discussion below). However, to identify a difference in quality scores of 3.5 points, we required at least 12 Level II studies (80% study power, alpha = 0.05). The more relevant comparison of the abridged quality scores that reflect the level of evidence criteria suggested that we would require at least 22 Level II studies. Given that only 3 Level II therapy studies have been published over the two-year period, it may require a decade to gain this additional information from the JBJS-A unless the Levels of Evidence Rating System is widely adopted by multiple orthopaedic journals. Therefore, our findings represent the current best estimate of association until more studies become available for comparison. Our study does, however, have a sufficient number of RCTs to observe variation in the study reporting quality scores. Since 2005, the JBJS-A has abandoned the uses of Level I and II subgroups; therefore, the relevance of analysing differences between Level Ia and Ib studies is limited. Our study described RCTs in one journal dedicated to one surgical field. Although this journal's scope is general orthopaedics, our findings are not generalisable to other surgical fields and journals.

### Previous literature

A previous review of published studies in The Journal of Bone and Joint Surgery 1988 through 2000 revealed a similar proportion (3%) of randomised trials compared with our current study (3.4%) [4]. The Cochrane Bone, Joint and Muscle Trauma Group's reporting quality assessment tool describes the following aspects of quality assessment which have previously been shown to be important in preventing bias [9]: allocation concealment, blinding, generation of allocation sequence, similarity of groups at baseline, description of outcomes, intention to treat analysis, and losses to follow-up. Currently, no consensus on the ideal checklist and scale for assessing methodological quality exists [9]. The number and variety of quality assessment scales that exist make it unclear as to how to achieve the best assessment [10,11]. The Levels of Evidence Rating System used by the JBJS-A can be qualified as one of these quality assessment scales. Summary scores (totals) should not be calculated, although it may be tempting to do so. The use of thresholds skews the direction of results and may lead to false conclusions in a meta-analyses [10]. Furthermore, Juni et al. discouraged the use of individual scales as absolute and objective measures of trial quality and noted "relevant methodological aspects should be identified, ideally a priori, and assessed individually" [10,18]. For example, the same criteria for blind assessment cannot be applied to drug and surgical trials, since, in the latter group, treatments are usually more difficult to conceal. Ideally, scales that are used to measure the quality of reporting of surgical trials should be tailored to the maximal possible quality, rather than to a unique gold-standard quality [10]. Therefore, the Cochrane Collaboration's handbook advises to describe aspects of critical appraisal separately and to avoid summarizing results [18]. Our findings confirm the variability of scores across each item of the Cochrane reporting quality assessment tool.

### Relevance of our findings

Despite the widely held belief that the Levels of Evidence Rating system categorizes studies by quality [5,6], our study suggests that this system, while reliable [7], may not be a valid tool for determining the quality of a study, as determined through the study reporting. As with any system, whether it is the Levels of Evidence Rating or the Cochrane reporting quality tool, the quality of study reporting is critical. The CONSORT statement was developed to help authors improve the reporting quality of RCTs [22]. In principle, this standardized scheme would explicitly require reporting of all features critical to the validity of a RCT and would require the presentation of results in a standard manner to improve clarity [4]. Use of the CONSORT statement is associated with improvements in the reporting quality of RCTs [22]. However, the reporting quality of RCTs in fracture care did not improve following the introduction of the CONSORT statement because many author's have not adopted the statement to guide their reporting [3]. Our findings further identify a lack of incorporation of the CONSORT statement in orthopaedic trials; only four studies (13%) were adequately reported with CONSORT guidelines. Journal editorial boards and assessors must continue to enforce high quality reporting of RCTs to allow an accurate assessment of the level of evidence and other study reporting quality measures.

### Implications for future research

This study was underpowered to explore the influence of reported statistical support, adherence to CONSORT guidelines, multi-centre studies, and sources of funding on the quality of reporting, direction of results, and magnitude of treatment effect size. Future studies are needed to explore any associations.

## Conclusion

Our findings suggest that readers should not assume that 1) studies labelled as Level I have high quality of reporting and 2) Level I studies have better reporting quality than Level II studies. Methodological safeguards should be addressed individually.

## Competing interests

RWP is supported, in part, by a Stichting Wetenschappelijk Onderzoek Orthopaedische Chirurgie Fellowship, Biomet Netherlands, Anna Fonds, Zimmer The Netherlands, Stryker The Netherlands, MSD The Netherlands, and a Nederlandse Vereniging voor Orthopedische Traumatologie Fellowship. MB is supported, in part, by a Canada Research Chair from the Canadian Institutes of Health Research.

The funding bodies were not involved in study design; not in the collection, analysis, and interpretation of data; not in the writing of the manuscript; and not in the decision to submit the manuscript for publication. The other authors did not receive funding.

## Authors' contributions

RWP and MB generated the study question. RWP, MB, and PAAS participated in the study design. RWP, RK, INS, and PAAS abstracted the data. RWP, MB, and INS participated in the data analysis, interpretation, and performed the statistical analysis. RWP and MB drafted the first version of the manuscript.

RWP, MB, PAAS, INS, RK, and KHL participated in drafting the subsequent versions of the manuscript and participated in the critical review of manuscript. All authors read and approved the final manuscript.

## Pre-publication history

The pre-publication history for this paper can be accessed here:



## Supplementary Material

Additional File 1 **The Cochrane Bone, Joint and Muscle Trauma Group tool for reporting of methodological quality**. The reporting quality of the included RCTs was scored with the Cochrane reporting quality assessment tool, which was devised by the Cochrane Bone, Joint and Muscle Trauma Group, formally known as the Musculoskeletal Injuries Group. This scoring scheme covers aspects of internal and external validity for the assessment of methodological quality. Click here for file

Additional file 2 **References to included studies**. Thirty-two RCTs that fit the eligibility criteria. Click here for file
