# Supplementary material for: Does a "Level I Evidence" rating imply high quality of reporting in orthopaedic randomised controlled trials?
Source: BMC Med Res Methodol. 2006 Sep 11;6:44. doi: 10.1186/1471-2288-6-44 (PMC1590046; doi:10.1186/1471-2288-6-44)
Supplement: Additional File 1 — The Cochrane Bone, Joint and Muscle Trauma Group tool for reporting of methodological quality. The reporting quality of the included RCTs was scored with the Cochrane reporting quality assessment tool, which was devised by the Cochrane Bone, Joint and Muscle Trauma Group, formally known as the Musculoskeletal Injuries Group. This scoring scheme covers aspects of internal and external validity for the assessment of methodological quality. [file 1471-2288-6-44-s1.doc]

# Additional files 1.

### The Cochrane Bone, Joint and Muscle Trauma Group score for reporting of methodological quality

*A. Was the assigned treatment adequately concealed prior to allocation?*

2= method did not allow disclosure of assignment.

1= small but possible chance of disclosure of assignment or unclear.

0= quasi-randomised or open list/tables.

Cochrane code: Clearly Yes = A; Not sure = B; Clearly No = C

B. Were the outcomes of participants who withdrew described and included in the analysis (intention to treat)?

2= withdrawals well described and accounted for in analysis.

1= withdrawals described and analysis not possible.

0= no mention, inadequate mention, or obvious differences and no adjustment.

*C. Were the outcome assessors blinded to treatment status?*

2= effective action taken to blind assessors.

1= small or moderate chance of unblinding of assessors.

0= not mentioned or not possible.

*D. Were the treatment and control group comparable at entry?*

2= good comparability of groups, or confounding adjusted for in analysis.

1= confounding small; mentioned but not adjusted for.

0= large potential for confounding, or not discussed.

*E. Were the participants blind to assignment status after allocation?*

2= effective action taken to blind participants.

1= small or moderate chance of unblinding of participants.

0= not possible, or not mentioned (unless double-blind), or possible but not done.

*F. Were the treatment providers blind to assignment status?*

2= effective action taken to blind treatment providers.

1= small or moderate chance of unblinding of treatment providers.

0= not possible, or not mentioned (unless double-blind), or possible but not done.

*G. Were care programmes, other than the trial options, identical?*

2= care programmes clearly identical.

1= clear but trivial differences.

0= not mentioned or clear and important differences in care programmes.

*H. Were the inclusion and exclusion criteria clearly defined?*

2= clearly defined.

1= inadequately defined.

0= not defined.

*I. Were the interventions clearly defined?*

2= clearly defined interventions are applied with a standardised protocol.

1= clearly defined interventions are applied but the application protocol is not standardised.

0= intervention and/or application protocol are poorly or not defined.

J. Were the outcome measures used clearly defined? (by outcome)

2= clearly defined.

1= inadequately defined.

0= not defined.

K. Were diagnostic tests used in outcome assessment clinically useful? (by outcome)

2= optimal.

1= adequate.

0= not defined, not adequate.

L. Was the surveillance active, and of clinically appropriate duration?

2= active surveillance and appropriate duration.

1= active surveillance, but inadequate duration.

0= surveillance not active or not defined.

A coding manual is available at the Cochrane website[15].
