# Supplementary material for: Does a "Level I Evidence" rating imply high quality of reporting in orthopaedic randomised controlled trials?
Source: BMC Med Res Methodol. 2006 Sep 11;6:44. doi: 10.1186/1471-2288-6-44 (PMC1590046; doi:10.1186/1471-2288-6-44)
Supplement: Additional file 2 — References to included studies. Thirty-two RCTs that fit the eligibility criteria. [file 1471-2288-6-44-s2.doc]

# Additional file 2.

### References to included studies

1. Horas U, Pelinkovic D, Herr G, Aigner T, Schnettler R. Autologous Chondrocyte Implantation and Osteochondral Cylinder Transplantation in Cartilage Repair of the Knee Joint: A Prospective, Comparative Trial. J Bone Joint Surg Am 2003;85(2):185-92.

2. Lehtonen H, Jarvinen TLN, Honkonen S, Nyman M, Vihtonen K, Jarvinen M. Use of a Cast Compared with a Functional Ankle Brace After Operative Treatment of an Ankle Fracture: A Prospective, Randomized Study. J Bone Joint Surg Am 2003;85(2):205-11.

3. Waters TS, Bentley G. Patellar Resurfacing in Total Knee Arthroplasty: A Prospective, Randomized Study. J Bone Joint Surg Am 2003;85(2):212-7.

4. Werber KD, Raeder F, Brauer RB, Weiss S. External Fixation of Distal Radial Fractures: Four Compared with Five Pins: A Randomized Prospective Study. J Bone Joint Surg Am 2003;85(4):660-6.

5. Wood K, Buttermann G, Mehbod A, Garvey T, Jhanjee R, Sechriest V. Operative Compared with Nonoperative Treatment of a Thoracolumbar Burst Fracture without Neurological Deficit: A Prospective, Randomized Study. J Bone Joint Surg Am 2003;85(5):773-81.

6. Leopold SS, Redd BB, Warme WJ, Wehrle PA, Pettis PD, Shott S. Corticosteroid Compared with Hyaluronic Acid Injections for the Treatment of Osteoarthritis of the Knee: A Prospective, Randomized Trial. J Bone Joint Surg Am 2003;85(7):1197-203.

7. DiGiovanni BF, Nawoczenski DA, Lintal ME, Moore EA, Murray JC, Wilding GE et al. Tissue-Specific Plantar Fascia-Stretching Exercise Enhances Outcomes in Patients with Chronic Heel Pain: A Prospective, Randomized Study. J Bone Joint Surg Am 2003;85(7):1270-7.

8. Mueller MJ, Sinacore DR, Hastings MK, Strube MJ, Johnson JE. Effect of Achilles Tendon Lengthening on Neuropathic Plantar Ulcers*: A Randomized Clinical Trial. J Bone Joint Surg Am 2003;85(8):1436-45.

9. Honl M, Dierk O, Gauck C, Carrero V, Lampe F, Dries S et al. Comparison of Robotic-Assisted and Manual Implantation of a Primary Total Hip Replacement: A Prospective Study. J Bone Joint Surg Am 2003;85(8):1470-8.

10. Caja VL, Piza G, Navarro A. Hydroxyapatite Coating of External Fixation Pins to Decrease Axial Deformity During Tibial Lengthening for Short Stature. J Bone Joint Surg Am 2003;85(8):1527-31.

11. Kim YH, Kim JS, Oh SH, Kim JM. Comparison of Porous-Coated Titanium Femoral Stems with and without Hydroxyapatite Coating. J Bone Joint Surg Am 2003;85(9):1682-8.

12. Koval KJ, Egol KA, Polatsch DB, Baskies MA, Homman JP, Hiebert RN. Tape Blisters Following Hip Surgery: A Prospective, Randomized Study of Two Types of Tape. J Bone Joint Surg Am 2003;85(10):1884-7.

13. The Canadian Orthopaedic Trauma Society. Nonunion Following Intramedullary Nailing of the Femur with and without Reaming. Results of a Multicenter Randomized Clinical Trial. J Bone Joint Surg Am 2003;85(11):2093-6.

14. Wang CJ, Wang JW, Weng LH, Hsu CC, Huang CC, Chen HS. The Effect of Alendronate on Bone Mineral Density in the Distal Part of the Femur and Proximal Part of the Tibia After Total Knee Arthroplasty. J Bone Joint Surg Am 2003;85(11):2121-6.

15. Cassidy C, Jupiter JB, Cohen M, Delli-Santi M, Fennell C, Leinberry C et al. Norian SRS Cement Compared with Conventional Fixation in Distal Radial Fractures. A Randomized Study. J Bone Joint Surg Am 2003;85(11):2127-37.

16. Brodner W, Bitzan P, Meisinger V, Kaider A, Gottsauner-Wolf F, Kotz R. Serum Cobalt Levels After Metal-on-Metal Total Hip Arthroplasty. J Bone Joint Surg Am 2003;85(11):2168-73.

17. Leung F, Chow SP. A Prospective, Randomized Trial Comparing the Limited Contact Dynamic Compression Plate with the Point Contact Fixator for Forearm Fractures. J Bone Joint Surg Am 2003;85(12):2343-8.

18. Wang CJ, Wang JW, Weng LH, Hsu CC, Huang CC, Yu PC. Prevention of Deep-Vein Thrombosis After Total Knee Arthroplasty in Asian Patients. Comparison of Low-Molecular-Weight Heparin and Indomethacin. J Bone Joint Surg Am 2004;86(1):136-40.

19. Kriegs-Au G, Petje G, Fojtl E, Ganger R, Zachs I. Ligament Reconstruction with or without Tendon Interposition to Treat Primary Thumb Carpometacarpal Osteoarthritis. A Prospective Randomized Study. J Bone Joint Surg Am 2004;86(2):209-18.

20. Swanik CB, Lephart SM, Rubash HE. Proprioception, Kinesthesia, and Balance After Total Knee Arthroplasty with Cruciate-Retaining and Posterior Stabilized Prostheses. J Bone Joint Surg Am 2004;86(2):328-34.

21. Knutsen G, Engebretsen L, Ludvigsen TC, Drogset JO, Grontvedt T, Solheim E et al. Autologous Chondrocyte Implantation Compared with Microfracture in the Knee. A Randomized Trial. J Bone Joint Surg Am 2004;86(3):455-64.

22. Faber FWM, Mulder PGH, Verhaar JAN. Role of First Ray Hypermobility in the Outcome of the Hohmann and the Lapidus Procedure. A Prospective, Randomized Trial Involving One Hundred and One Feet. J Bone Joint Surg Am 2004;86(3):486-95.

23. Buttermann GR. Treatment of Lumbar Disc Herniation: Epidural Steroid Injection Compared with Discectomy. A Prospective, Randomized Study. J Bone Joint Surg Am 2004;86(4):670-9.

24. Paoloni JA, Appleyard RC, Nelson J, Murrell GAC. Topical Glyceryl Trinitrate Treatment of Chronic Noninsertional Achilles Tendinopathy. A Randomized, Double-Blind, Placebo-Controlled Trial. J Bone Joint Surg Am 2004;86(5):916-22.

25. Aglietti P, Giron F, Buzzi R, Biddau F, Sasso F. Anterior Cruciate Ligament Reconstruction: Bone-Patellar Tendon-Bone Compared with Double Semitendinosus and Gracilis Tendon Grafts. A Prospective, Randomized Clinical Trial. J Bone Joint Surg Am 2004;86(10):2143-55.

26. Ogden JA, Alvarez RG, Levitt RL, Johnson JE, Marlow ME. Electrohydraulic High-Energy Shock-Wave Treatment for Chronic Plantar Fasciitis. J Bone Joint Surg Am 2004;86(10):2216-28.

27. Aigner C, Windhager R, Pechmann M, Rehak P, Engeleke K. The Influence of an Anterior-Posterior Gliding Mobile Bearing on Range of Motion After Total Knee Arthroplasty. A Prospective, Randomized, Double-Blinded Study. J Bone Joint Surg Am 2004;86(10):2257-62.

28. Kay RM, Rethlefsen SA, Fern-Buneo A, Wren TAL, Skaggs DL. Botulinum Toxin as an Adjunct to Serial Casting Treatment in Children with Cerebral Palsy. J Bone Joint Surg Am 2004;86(11):2377-84.

29. Tsumaki N, Kakiuchi M, Sasaki J, Ochi T, Yoshikawa H. Low-Intensity Pulsed Ultrasound Accelerates Maturation of Callus in Patients Treated with Opening-Wedge High Tibial Osteotomy by Hemicallotasis. J Bone Joint Surg Am 2004;86(11):2399-405.

30. Strohm PC, Muller CA, Boll T, Pfister U. Two Procedures for Kirschner Wire Osteosynthesis of Distal Radial Fractures. A RANDOMIZED TRIAL. J Bone Joint Surg Am 2004;86(12):2621-8.

31. Jirarattanaphochai K, Saengnipanthkul S, Vipulakorn K, Jianmongkol S, Chatuparisute P, Jung S. Treatment of de Quervain Disease with Triamcinolone Injection with or without Nimesulide. A RANDOMIZED, DOUBLE-BLIND, PLACEBO-CONTROLLED TRIAL. J Bone Joint Surg Am 2004;86(12):2700-6.

1. Bolognesi MP, Pietrobon R, Clifford PE, Vail TP. Comparison of a Hydroxyapatite-Coated Sleeve and a Porous-Coated Sleeve with a Modular Revision Hip Stem. A Prospective, Randomized Study. J Bone Joint Surg Am 2004;86(12):2720-5.
